# Current state of female pediatric urologists at Societies for Pediatric Urology fellowship accredited programs

**DOI:** 10.3389/fruro.2023.1104597

**Published:** 2023-01-27

**Authors:** Natalie Armon, Nora Thompson, Daniel DaJusta, Molly Fuchs, Christina B. Ching

**Affiliations:** ^1^ Department of Pediatric Urology, Nationwide Children’s Hospital, Columbus, OH, United States; ^2^ Kidney and Urinary Tract Center, Nationwide Children’s Hospital, Columbus, OH, United States; ^3^ Center for Research Services, Nationwide Children’s Hospital, Columbus, OH, United States

**Keywords:** female, pediatric urology, gender equity, academic medicine, fellowship and training

## Abstract

**Purpose:**

Gender inequities in medicine exist. Urology is a male dominated surgical specialty, with recent census data showing females represent only 10.9% of the workforce. We aimed to characterize the composition of female pediatric urologists across the Societies for Pediatric Urology (SPU) pediatric urology fellowship accredited programs, with particular attention to academic promotion and leadership positions.

**Methods:**

In January 2023, we reviewed the official websites of the 27 pediatric urology programs listed on the SPU website as fellowship accredited programs. We identified pediatric urology surgery attendings, their gender, academic title, and if they were named a director of an internal program or had a hospital leadership position. We identified the program chief and fellow/resident program director. This data was associated with years in practice.

**Results:**

Females represented 27.4% of pediatric urology surgical attendings. Four programs (14.8%) had no female attendings. Female staff were in practice a median shorter time than that of males (6 vs. 16 years, p<0.0001). A significantly higher proportion of females were assistant professors (62.2 vs. 35.2%; p=0.0041) while a significantly higher proportion of men were professors (37.0 vs. 18.9%; p=0.0421). Only one program (3.7%) had a female department/division chief. There was no difference between genders regarding being named a director of a program and/or having an identified hospital position of leadership. Female professors had been in practice a significantly shorter time than male professors (p=0.0003); women with an internal or hospital leadership position had also been in practice a significantly shorter time than males (p<0.001).

**Conclusions:**

Females are represented more in SPU pediatric urology fellowship accredited programs than the overall urology workforce. Fewer female attendings are professors compared to male attendings; however, differences in promotion could be impacted by female attendings being earlier in their career. Hopefully with time, we will see more equal representation amongst genders in pediatric urology programs overall, but especially where we are training the next generation.

## Introduction

Urology has historically been a male dominated field, with the most recent specialty census data from 2021 finding only 10.9% of practicing urologists are female (1). Despite this, there is encouraging data to suggest the gender gap is closing, with a 17-fold increase in the number of female urology residents since 1978: women filled just 1.9% of urology residency spots in 1978 (2) while 2021 urology residency statistics have females representing 33.7% of matched urology residents (3). Further projection models suggest the number of female urologists will continue to steadily increase over the next 40 years (4).

Despite the increase of female urologists, equal gender representation in leadership positions is still lacking, with ramifications in academic promotion. Women hold 2.5% of advanced leadership positions (i.e. department chairs or members of the American Board of Urology and the Society of Academic Urologists) and 10% of leadership positions in urology committees (5). In a 2017 study of 124 U.S. urology residency programs, women comprised 3.3% of chairs and 7.9% of division directors (6). Concordant with these findings is that fewer women attain senior academic ranking, with another 2017 study of academic urologic institutions reporting male urologists were twice as likely to advance to full professorship compared to their female counterparts (33% vs 12%) (7).

We sought to provide an up-to-date view of the landscape of gender representation in the Societies for Pediatric Urology (SPU) pediatric urology fellowship accredited programs which we feel represent highly visible academic pediatric urology programs responsible for training the next generation of pediatric urologist. We identified female pediatric urologists in these programs, paying particular attention to academic promotion and leadership positions. While the 2017 study of U.S. urology residency programs did identify pediatric urology as one of the top specialties with female representation of division directors (9.8%) (6), to our knowledge, there has not been a specific characterization of female providers in pediatric urology until now. We hypothesized that female staff in pediatric urology would have fewer leadership roles and be less likely to achieve academic promotion than their male counterparts.

## Materials and methods

We reviewed the official websites of those programs listed on the SPU website (spuonline.org) as pediatric urology fellowship accredited programs between January 7-8, 2023. There were a total of 27 programs (see **Supplemental Figure**). We looked for the webpage that listed the staff members associated with each program. Upon review of the site, we identified staff gender, academic title, and if they were named a director of an internal program or held an obvious hospital leadership position (e.g. chief of staff). Specifically, we looked for the words “director” and/or “chief” in their titles or biographies. We similarly identified the pediatric urology department or division chief and pediatric urology fellowship director. We also looked to see if any staff were listed as a resident program director if the program had a residency.

Information regarding the date staff graduated from pediatric urology fellowship or completed last training was sought. This was first evaluated on the program website. If such information was not available, however, a broader google search was performed to try and identify information regarding training/fellowship program graduation *via* common websites like doximity.com and https://health.usnews.com /doctors. From this, we calculated years in practice by subtracting the identified year from the year 2022.

Across all programs evaluated, we compared the gender of staff members and their years in practice. We calculated the percent outcome, such as those of a specific academic title and those named director of an internal program and/or in a hospital leadership position, within each gender for comparison between genders. Lastly, we compared years in practice within each academic title and of those with a directorship role across genders as a way to evaluate the time to achievement of these milestones. Normal distribution within a category was tested using D’Agostino & Pearson test. Parametric variables were compared using unpaired t-test and non-parametric variables were compared using Mann-Whitney test. Distribution of genders for academic title was compared by chi-square test. A p-value of <0.05 was considered significant.

This study was reviewed by our Institutional Review Board and not deemed research involving human subjects (STUDY00003055).

## Results

**Figure 1:**
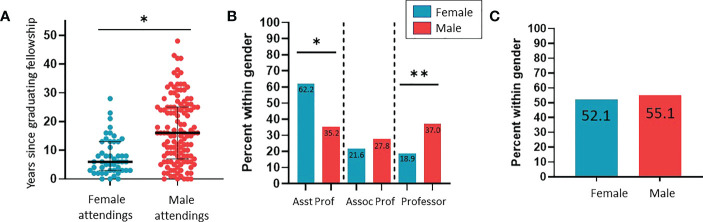
Characterization of career findings between genders of pediatric urology attendings at SPU fellowship accredited programs. **(A)** Years since graduating fellowship training (*p<0.0001). **(B)** Comparison across gender of those with professor and assistant professor academic titles (*p=0.0243; **p=0.0258). **(C)** Comparison across gender of those with “director” or “chief” in their listed titles.

All SPU accredited programs had program websites for review. Female pediatric urologists represented 27.4% (48 of 175) of all pediatric urology surgical attendings listed on the websites of these programs. Four of the programs (14.8%) had no female attendings listed. Looking at years since training ended, female staff had been in practice a significantly shorter time than male counterparts (median 6 years vs. 16 years, p<0.0001) (**Figure 1A**).

**Figure 2:**
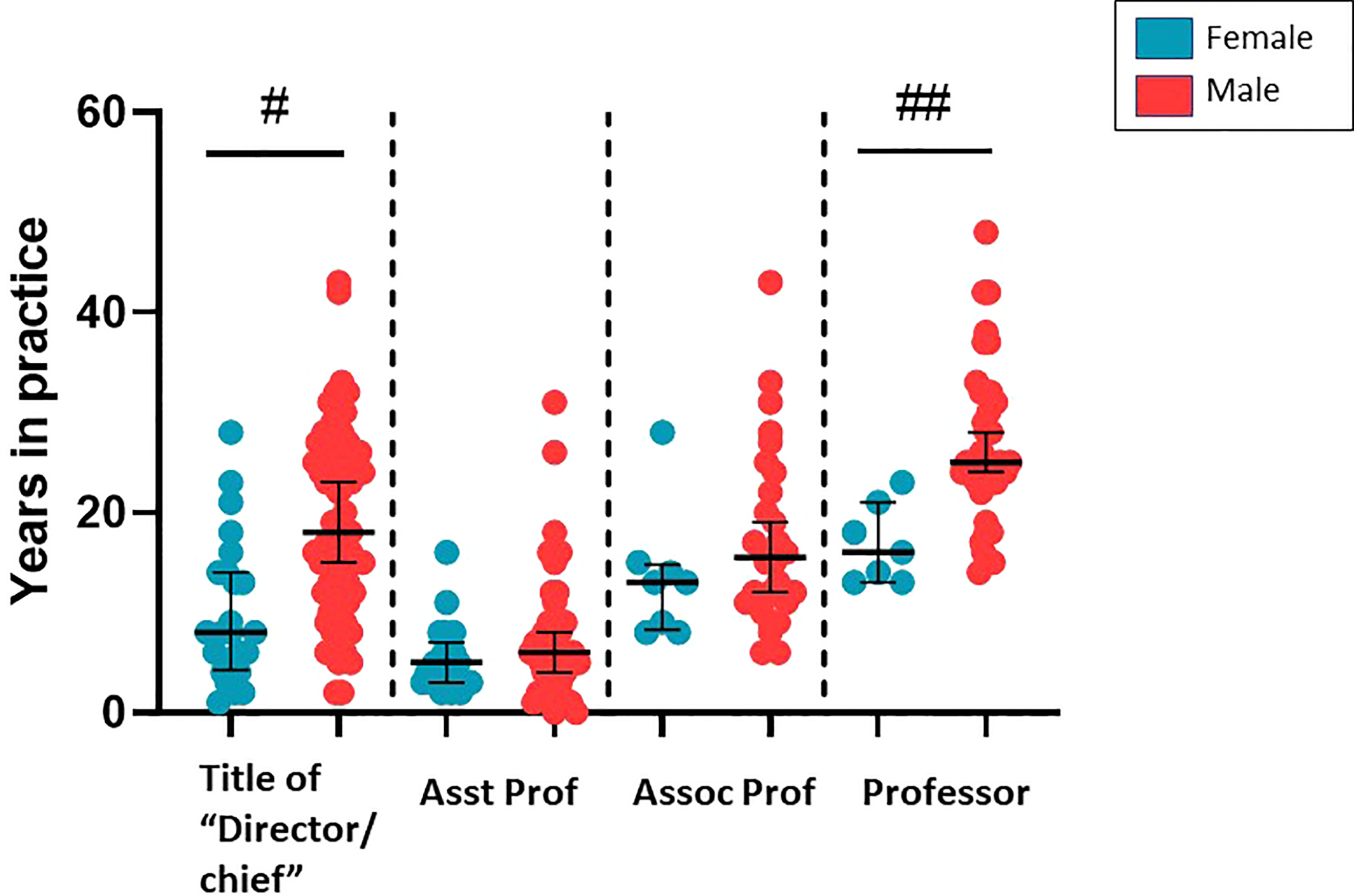
Comparison of having a title of director/chief or academic title and years in practice by gender (#p<0.0001; ##p=0.0003).

We investigated the prevalence of female leadership amongst programs. Only 1 program had a female chief (3.7%). Eight (29.6%) females were fellowship program directors. Only 4 programs had a pediatric staff listed as the urology residency program director of which the majority were female (75%). We were able to identify an academic title on the program website for 37 of the 48 (77.1%) women identified and 108 of the 127 (85.0%) men identified. Women without an identifiable academic title had been in practice a significantly shorter period of time than males without (median 3.1 vs. 14.8 years; p=0.0060). Of those with a listed academic title on their program website, significantly more females were assistant professor and significantly more males were professors (**Figure 1B**). There was no significant difference between genders regarding “director” or “chief” in their listed titles (p=0.4624) (**Figure 1C**). Female directors of an internal program and/or in a hospital leadership position had been in practice a significantly shorter period of time than males (median 10.0 years vs. 19.0 years; p<0.0001). Similarly female professors had been in practice a significantly shorter period of time than male professors (median 16.0 years vs. 25.0 years; p=0.0003) (**Figure 2**).

## Discussion

We are the first to report the specific representation of female staff urologists in pediatric urology programs and identify their roles in leadership positions and their academic rank. We chose to review the pediatric urology programs that have accredited fellowship programs by the SPU as we felt it important to identify the demographics of those training the next generation of pediatric urologists. We found some reassuring statistics regarding gender diversity within a representation of pediatric urology fellowship programs. Women made up 27.4% of pediatric urology surgical attendings in these programs, which is over double that reported generally for practicing urologists (10.9%) (1). These female attendings had been in practice a significantly shorter amount of time than their male counterparts, reflecting an overall change in gender demographics in the urology workforce (8). According to the American Urological Association’s 2021 census, women comprise 23.2% of practicing urologists under 45 years old but only 7.0% of those over 65 years old (1). Increased gender diversity in pediatric urology is likely due to these overall changes in composition of individuals entering urology and a high volume of women entering the subspecialty of pediatric urology (1, 2, 7, 9–11).

Despite our encouraging discovery regarding general female provider representation in pediatric urology, we found less promising evidence of female providers having major pediatric urology departmental leadership positions. Only one (3.7%) of SPU accredited pediatric urology fellowship programs had a female department/division chief which is consistent with other data showing women hold only 3.3% of urology department chairs (6). Even then, urology appears to lag significantly behind that of reports of female leadership representation in academic medicine overall, where women hold 29% of department chief positions (12). Interestingly, our findings were consistent with prior research demonstrating female chief leadership does not necessarily increase gender diversity or female leadership within a program (13), with the one identified female lead program lacking additional female attendings and consequently other female leaders. Yet one can assume that the lack of female departmental/division leaders contributes to a lack of senior level female mentorship that can be important for physician retention, job satisfaction and promotion (14).

Compared to chief positions, the numbers are more favorable of women holding other positions of leadership within our search. There was no difference between male and female attendings with listed internal leadership positions. When evaluating females in educational positions, our findings show a higher representation of women holding pediatric urology fellowship directorships (29.6%) than that reported more broadly for all urology subspecialty fellowships (9.4%) (6). Female leadership representation in the educational sphere has some mixed interpretations. It has been stressed that female program directorship positions provide more mentorship opportunities to female trainees and could lead to better recruitment of female residents and fellows into respective programs. A recent study of internal medicine programs, however, did not find this to be the case with no association found between presence of a female program director and number women trainees (15); this has of yet to be investigated in pediatric urology, however. It has been suggested that an apparent discrepancy between representation of women in program directorship positions compared to department/division chief positions is a result of women generally being guided toward more clinic-educator career tracks rather than traditional research-based tracks that have a higher likelihood of major departmental leadership advancement and thus promotion (16).

We were most interested in the impact gender might have on academic promotion in the pediatric urology sphere. Previous research has found large discrepancies in the academic titles of male and female urologists (7, 17). Our study similarly found women to be significantly of more junior rank (e.g. assistant professor) when compared to men who were more commonly full professors. When looking at the years of practice between genders with these titles, however, we found that female professors were earlier in their career than males with the same title. This appears to be an improvement from prior work evaluating the timeline of promotion between males and females in academic urology. Breyer et al. found women require 1.2 years longer than men for promotion from assistant to associate professor; and that men are 3 times more likely to be promoted in under 4 years (17). While we did not identify the time of academic promotion in order to evaluate this ourselves, our findings are overall encouraging that the differences we see in academic title may be a factor of female pediatric urologists being overall in practice a shorter amount of time. With time, we would hope that some of the representation amongst higher academic rank will equilibrate as female staff have more time to obtain the chance for promotion.

We are still far off from normalizing the presence of women in the field of urology and creating an inclusive culture. In a recent survey by Haslam et. al, male urologists reported higher scores for work culture, support from leadership, freedom from gender bias, and gender equality in career development than women. In this study, they found male urologists believed gender equality has already been achieved and even seem resistant to the conversation (8). In contrast, Jackson et al. found in a survey of female urology residents that 36% reported inappropriate treatment by male staff members and 22% mentioned sexual harassment (18). While these are overt demonstrations of aggression against females, seemingly more trivial acts can alienate and minimize the presence of women in the specialty, such as colleagues calling them by their first name while referring to a male counterpart as “Dr” (8). Patients themselves can contribute to the problem, with a survey of female urology residents finding 60% had experienced a male patient refusing their care and 29% reporting inappropriate treatment by male patients (18). Interestingly, patient refusal may contribute to female urologists entering subspecialties with less adult male patients, such as pediatric urology.

Our investigation demonstrates some encouraging trends with signs of increasing female numbers and representation in certain leadership roles and seemingly more appropriate academic promotion than anticipated. Work still exists, however, to improve the working space for females within urology overall. We should note that this study is limited by dependence on information being accurate and up to date on the internet. In addition, our study is limited to programs with SPU accredited pediatric fellowships, biasing to more academic practices that might represent a more diverse faculty according to important strides being made in the equity, diversity, and inclusion of program faculty. As a result, our outcomes might not be reflective of programs without a pediatric fellowship program and thus pediatric urology as a whole. We also were unable to evaluate external leadership programs which would be interesting in order to evaluate the place of female pediatric urologists on a national and international level. It will remain important to continue to keep a pulse on the future of females in pediatric urology and recommend periodic review and updates.

## Data availability statement

The raw data supporting the conclusions of this article will be made available by the authors, without undue reservation.

## Author contributions

NA and CC contributed to concept origination, paper outlining, and writing/editing. MF was involved in concept origination and significant editing. NT and DD were involved in data analysis and significant editing. All authors contributed to the article and approved the submitted version.

## Supplementary material

Supplementary Figure List of Societies for Pediatric Urology accredited Pediatric Urology Fellowship Programs as listed on their official website (spuonline.org) (as of Jan 9, 2023).

## References

[1] American Urological Association. The state of the urology workforce and practice in the United States (2021) Available at: https://www.auanet.org/research-and-data/aua-census/census-results.

[2] Halpern JA Lee UJ Wolff EM Mittal S Shoag JE Lightner DJ. Women in Urology Residency, 1978-2013: A Critical Look at Gender Representation in Our Specialty. Urology (2016) 92:20–5. doi: 10.1016/j.urology.2015.12.092 26952568

[3] American Urological Association. 2021 Urol Residency Match Statistics. (2021). Available at: https://www.auanet.org/documents/education/specialty-match/2021-Urology-Residency-Match-Statistics.pdf

[4] Nam CS Daignault-Newton S Kraft KH Herrel LA. Projected US Urology Workforce per Capita, 2020-2060. JAMA Netw Open (2021) 4(11):e2133864. doi: 10.1001/jamanetworkopen.2021.33864 34783827 PMC8596195

[5] Cancian M AL Thavaseelan S. The representation of women in urological leadership. Urol Practice. (2018) 5(2):228–32. doi: 10.1016/j.urpr.2017.03.006 37300232

[6] Han J Stillings S Hamann H Terry R Moy L. Gender and subspecialty of urology faculty in department-based leadership roles. Urology. (2017) 110:36–9. doi: 10.1016/j.urology.2017.07.044 28802569

[7] Mayer EN Lenherr SM Hanson HA Jessop TC Lowrance WT. Gender differences in publication productivity among academic urologists in the united states. Urology. (2017) 103:39–46. doi: 10.1016/j.urology.2016.12.064 28232174 PMC5532805

[8] Haslam RE Collins A Martin LH Bassale S Chen Y Seideman CA. Perceptions of gender equity in pediatric urology. J Pediatr Urol. (2021) 17(3):406 e1– e7. doi: 10.1016/j.jpurol.2021.01.011 33558178

[9] American Urological Association. The state of the urology workforce and practice in United States (2020). Available at: https://www.auanet.org/research-and-data/aua-census/census-results.

[10] Porten SP Gaither TW Greene KL Baradaran N Anger JT Breyer BN. Do women work less than men in urology: Data from the American urological association census. Urology. (2018) 118:71–5. doi: 10.1016/j.urology.2018.04.015 29723591

[11] Spencer ES Deal AM Pruthi NR Gonzalez CM Kirby EW Langston J. Gender differences in compensation, job satisfaction and other practice patterns in urology. J Urol. (2016) 195(2):450–5. doi: 10.1016/j.juro.2015.08.100 PMC5004345 26384452

[12] Association of American Medical Colleges. 2018-2019 The state of women in academic medicine: exploring pathways to equity. (2020). Available at: https://www.aamc.org/data-reports/data/2018-2019-state-women-academic-medicine-exploring-pathways-equity.

[13] Kennedy GE Bergstresser SL Rakestraw SL Novak Z Corey B Chen H. Does chair of surgery gender influence divisional or residency program director gender diversity? J Surg Res (2021) 267:224–8. doi: 10.1016/j.jss.2021.05.026. 34157491

[14] Farkas AH Bonifacino E Turner R Tilstra SA Corbelli JA. Mentorship of women in academic medicine: A systematic review. J Gen Intern Med (2019) 34(7):1322–9. doi: 10.1007/s11606-019-04955-2 PMC6614283 31037545

[15] Medepalli K Purdon S Bade RM Glassberg MK Burnham EL Gershengorn HB. Association of women leaders with women program director and trainee representation across US academic internal medicine. J Gen Intern Med (2022) 38(1):57–66. doi: 10.1007/s11606-022-07635-w PMC9126637 35604632

[16] Khan MS Usman MS Siddiqi TJ Ayub MT Fatima K Acob C. Women in leadership positions in academic cardiology: A study of program directors and division chiefs. J Womens Health (Larchmt). (2019) 28(2):225–32. doi: 10.1089/jwh.2018.7135 30596542

[17] Breyer BN Butler C Fang R Meeks W Porten SP North AC. Promotion disparities in academic urology. Urology. (2020) 138:16–23. doi: 10.1016/j.urology.2019.10.042 31917291

[18] Jackson I Bobbin M Jordan M Baker S. A survey of women urology residents regarding career choice and practice challenges. J Womens Health (Larchmt). (2009) 18(11):1867–72. doi: 10.1089/jwh.2008.1236 19951224

