# Supplementary figures and images for: Current state of female pediatric urologists at Societies for Pediatric Urology fellowship accredited programs
Source: Front Urol. 2023 Jan 27;3:1104597. doi: 10.3389/fruro.2023.1104597 (PMC12327338; doi:10.3389/fruro.2023.1104597)

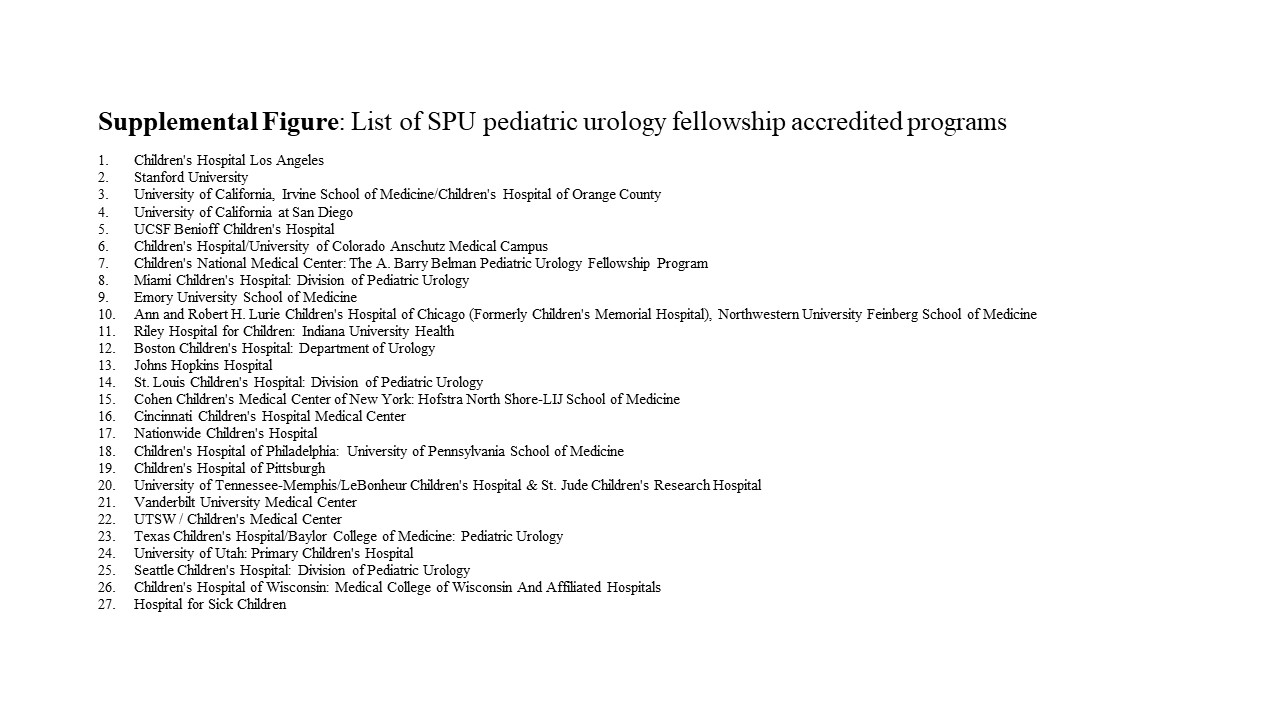

Supplement: Supplementary Figure — List of Societies for Pediatric Urology accredited Pediatric Urology Fellowship Programs as listed on their official website (spuonline.org) (as of Jan 9, 2023). [file Image_1.jpeg]
